# Effectiveness of Different Analytical Methods for the Characterization of Propolis: A Case of Study in Northern Italy

**DOI:** 10.3390/molecules25030504

**Published:** 2020-01-23

**Authors:** Radmila Pavlovic, Gigliola Borgonovo, Valeria Leoni, Luca Giupponi, Giulia Ceciliani, Stefano Sala, Angela Bassoli, Annamaria Giorgi

**Affiliations:** 1 Centre of Applied Studies for the Sustainable Management and Protection of Mountain Areas (CRC Ge.S.Di.Mont.), University of Milan, Via Morino 8, 25048 Edolo (BS), Italy; radmila.pavlovic1@unimi.it (R.P.); gigliola.borgonovo@unimi.it (G.B.); valeria.leoni@unimi.it (V.L.); giulia.ceciliani@unimi.it (G.C.); stefano.sala1@unimi.it (S.S.); angela.bassoli@unimi.it (A.B.); anna.giorgi@unimi.it (A.G.); 2 Department of Food, Environmental and Nutritional Sciences (DEFENS), University of Milan, Via Celoria 2, 20133 Milan, Italy; 3 Department of Agricultural and Environmental Sciences - Production, Landscape, Agroenergy (DISAA), Via Celoria 2, 20133 Milan, Italy

**Keywords:** propolis, poplar, HPLC–Q-Exactive-Orbitrap*^®^*–MS analysis, phenolic glycerides

## Abstract

Propolis is used as folk medicine due to its spectrum of alleged biological and pharmaceutical properties and it is a complex matrix not still totally characterized. Two batches of propolis coming from two different environments (plains of Po Valley and the hilly Ligurian–Piedmont Apennines) of Northern Italy were characterized using different analytical methods: Spectrophotometric analysis of phenols, flavones and flavonols, and DPPH radical scavenging activity, HPLC, NMR, HSPME and GC–MS and HPLC–MS Orbitrap. Balsam and moisture content were also considered. No statistical differences were found at the spectrophotometric analysis; balsam content did not vary significantly. The most interesting findings were in the VOCs composition, with the Po Valley samples containing compounds of the resins from leaf buds of *Populus nigra* L. The hills (Appennines) samples were indeed characterize by the presence of phenolic glycerides already found in mountain environments. HPLC–Q-Exactive-Orbitrap*^®^*–MS analysis is crucial in appropriate recognition of evaluate number of metabolites, but also NMR itself could give more detailed information especially when isomeric compounds should be identified. It is necessary a standardized evaluation to protect and valorize this production and more research on propolis characterization using different analytical techniques.

## 1. Introduction

Propolis, or bee glue, is a natural wax-like resinous substance found in beehives where it is used by honeybees as cement and to seal cracks or open spaces [1]. It is used by bees as well to prevent contamination inside the hive by bacteria, viruses or parasites because of its antiseptic effect; as well as to cover intruders who died inside the hive in order to avoid their decomposition [2]. Many comparison studies have now validated the theory that propolis is collected by honeybees from tree buds or other botanical sources in the North Temperate Zone, which extends from the Tropic of Cancer to the Arctic Circle [3]. The best sources of propolis are species of poplar, willow, birch, elm, alder, beech, conifer, and horse-chestnut trees [3]. Its color varies from green to brown and reddish, and the characteristic of each different type of propolis is dependent of some factors as e.g., plant source and edaphoclimatic conditions [4]. The word propolis derives from Hellenistic Ancient Greek *pro-* for or in defence, and *polis* city. There are records suggesting the use of it by ancient Egyptians, Persians, and Romans [5]. 

Recent studies confirmed the many properties of propolis: research over the last two to three decades has further exposed the wide potential of propolis, particularly its biological applications. Applications like anti-carcinogenic [6], anti-protozoan [7], anti-inflammatory [8], antioxidant [9], immunestimulating [10], antiviral [11], anti-tumor [10], hepato-protective [12], antifungal [13], and antibacterial activity [14], so it has been the subject of increasing scientific interest due to its diverse range of bio-medical properties. 

Modern herbalists recommend this bee product for its beneficial properties to increase the natural resistance of human organisms [14]. Today propolis is currently used as a popular remedy and is available in the form of capsules (either in pure form or combined with aloe gel and rosa canina or pollen), as an extract (hydroalcholic or glycolic), as a mouthwash (combined with melissa, sage, mallow andyor rosemary), in throat lozenges, creams, and in powder form (to be used in gargles or for internal use once dissolved in water). It is also available commercially as purified product in which the wax has been removed. Propolis is also claimed to be useful in cosmetics and as a constituent of health foods. Current opinion is that the use of standardized preparations of propolis is safe and less toxic than many synthetic medicines [15].

It is therefore to mention that still there are not quality standard for this product [16]. A European Community report states that, the economic value of propolis is difficult to measure because it has no legal definition and is not a registered product (Evaluation of CAP measures for the apiculture sector, final report, July 2013). Scientific research regarding its chemical composition and biological activity started only about 30 years ago [17]. Since bees use the natural available vegetation to create propolis, there is a high variability in the composition. 

It should be kept in mind, that bees collect their products for own benefit in the first place, and human beings are taking advantages of their hard work. If we take care to place the apiary in a location rich in food and material sources they need, then we have healthy bees contributing to pollination and biodiversity maintaining and other secondary aspects derived from these [16]. The role of bees in marginal ecosystems and agroecosystems is very important, and it is also essential to discriminate if the location of the apiary can influence the characteristics of this important bee product. Various factors give rise to the chemical complexity of propolis, for example, phyto-geographical origin, time of collection, and type of bees foraging. Complex chemical composition of propolis is the most important reason for many of the analytical challenges [18]. For what concerns phyto-geographical origin, chromatographic fingerprints are a valuable analytical method to identify different parts of plants [19].

Propolis is one of the most fascinating bee products, for sure a key factor of the success of the important macro-organism of the beehive, and its chemical complexities pose a great challenge to understanding content and percentage uniformity, and the connected biological activity. Complex chemical composition especially polarity of constituents makes it difficult to apply a single analytical technique vis a vis standardization even in today’s era of very advanced techniques like High Performance Liquid Chromatography (HPLC), Liquid Chromatography associated with Mass Spectrometry (LC–MS/MS), Liquid Chromatography-High Resolution Mass Spectrometry (LC–HRMS), Gas Chromatography associated with Mass Spectrometry and Solid Phase Microextraction (SPME–GC–MS), and Nuclear Magnetic Resonance (NMR). Propolis is prepared primarily as alcoholic extract and therefore the maceration in alcohol is the most common extraction method used also for experimental purposes. Spectrophotometry, especially the Folin–Ciocalteu method, is the most widely used for the routine determination of total content of phenols and certain groups of flavonoids in propolis. However, other spectrophotometry methodologies have also been widely applied. For example, 1,1-diphenyl-2-picrylhydrazyl (DPPH) is one of the widely used method in evaluation of the antioxidant activity of propolis [20]. 

The quantification of individual compounds shows a significant discrepancy in the results reported in the bibliography about total phenolic content [21]. Very often, phenolic acids and individual compounds are not quantified. This is mainly due to the difference in the reference standards chosen for the construction of the calibration curves necessary to express the quantitative result [22].

Chromatographic methods, especially HPLC, are used for the separation and quantification of the specific constituent compounds of the phenolic profile, although they are not recommended as routine procedures due to their high cost [23,24] and, of course, to the complexity of the propolis matrix. 

Advanced techniques as HPLC–high resolution mass spectrometry (HRMS) accompanied with a metabolomic approach could be a sufficiently descriptive method as it is able to detect the biomarkers that could be used as indicators of authentication. For the in-depth characterization of propolis recently developed analytical platforms based on NMR technique has been proved suitable for defining some a whole series of isomeric compounds found in propolis [25].

In addition to phenolics, another important class of propolis constituents is represented by volatile compounds [26,27,28]. Solid phase microextraction (SPME) represents a reliable tool for the analysis of volatile organic compounds [29,30] and eliminates most drawbacks to extracting organics, including high cost and excessive preparation time. SPME is a simple and fast modern tool used to characterize the volatile fraction of medicinal plants [31] and foods [29] and offers a valid alternative to HD for gas chromatographic analysis of essential oils from different sources [31]. For what concern propolis, SPME coupled with GC–MS can avoid the loss and degradation of volatile constituents that happen instead with HD (Hydro Distillation), very often used for the characterization of propolis volatiles [32,33].

Taking into account above elaborated considerations, the primary aim of this study was indeed to characterize two propolis produced in a hilly (Ligurian–Piedmont Apennines) and plain areas (Po Valley) of Northern Italy using diverse analytical approaches starting from basic ones (spectrophotometric analysis) to reach those more advanced such as HPLC, LC–HRMS, NMR, and SPME–GC–MS in order to evaluate the information that each of those method can provide for the characterization of propolis.

## 2. Results

### 2.1. Balsam and Moisture Content, Total Phenols, Flavones and Flavonols Content, and Scavenging Activity 

In Table 1 is reported the composition of propolis in balsam and the moisture content. Antioxidant content and the DPPH radical scavenging activity are also reported, measured as described in materials and methods. The results are expressed in mg/g and agree with previous research [21]. Balsam content was found higher in the hills’ samples (75.92 ± 4.92%, while in the plains samples was 63.94 ± 12.86%) but not significantly different according the statistical analysis. Very similar mean value (0.74 ± 0.38% for the hills batch and 0.69 ± 0.52% for the six samples collected in the plains) was found for the moisture content.

The total phenols, calculated as Gallic Acid Equivalents (mg GAE/g), the total Flavones and flavonols were determined using aluminum chloride and expressed as quercetine equivalent (mg QE/g) and the DPPH radical scavenging activity did not vary significantly between the hills and plains batches: the mean value of total flavones and flavonols was 32.14 ± 4.38 mg/g for the hills samples and 26.91 ±4.31 mg/g for the plains, the mean value of total phenol was 242.42 ± 11.67 for the hill samples and 236.32 ± 40.92 mg/g for the plains and this results were reflected by a very similar DPPH radical scavenging activity mean values (45.01 ± 1.39 for the hills and 46.44 ± 0.96 for the plains).

### 2.2. High-Performance Liquid Chromatography (HPLC) Analysis 

**Figure 1:**
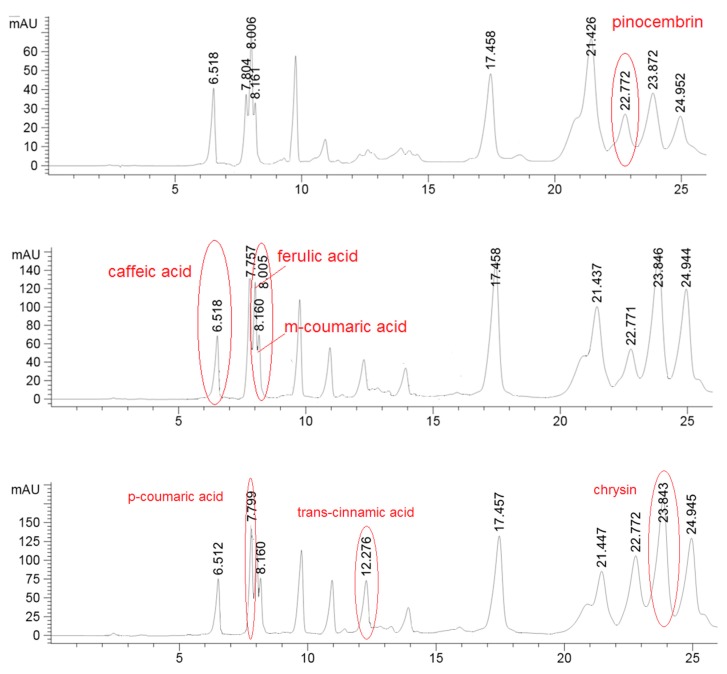
Detection of pinocembrin in one hills sample at 375 nm; detection of caffeic acid, ferulic acid and *m*-coumaric acid in one hills sample at 325 nm; detection of *p*-coumaric acid, *trans*-cinnamic acid and chrysin in one hills sample at 295 nm.

The HPLC analysis was performed in order to obtain the preliminary phenolic/flavonoids profile, using three different UV wavelength that were used for better identification of the compounds. For example, pinocembrin was detected on 375 nm, phenolic acids caffeic, m-coumaric and ferulic were monitored on 325 nm while p-coumaric and trans-cinammic acids along with chrysin were registrated at 295 nm (Figure 1). The quantity did not result significantly different for caffeic acid, chrysin and pinocembrin (Table 2). In hill samples, instead, it was found a considerably higher quantity of p-coumaric acid, ferulic acid, *m*-coumaric acid while the amount of *trans*-cinnamic acid was higher in the plain samples batch. 

### 2.3. Propolis Volatile Compounds

**Figure 2:**
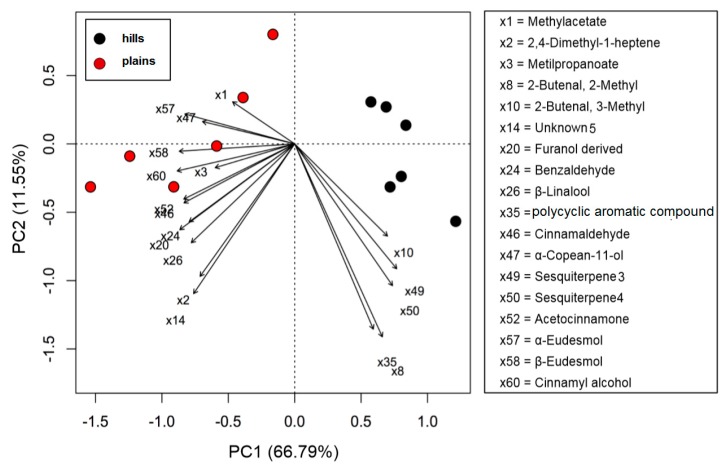
PCA biplot of volatile compounds (VOCs) of propolis samples collected in hills (Ligurian–Piedmont Apennines) and plains (Po Valley).

For what concerns the volatiles composition, a higher quantity of volatile compounds (VOCs) was found in the plains batches than in the hills (total VOCs of 415 µg/g for the hills and 502 µg/g for the plains), with a corresponding higher content of terpenes and terpenoids (85 µg/g on average for plains samples and 65 for hills samples). Hills and plains samples contained the same compounds, that varied only quantitatively for 18 compounds on the 60 compounds recognized. In the plains it was found a significantly higher quantity of methyl-acetate, 2,4-dimethyl-1-heptene, methyl propanoate, and benzaldehyde. The quantity was significantly higher β-Linalool, cinnamaldehyde, α-copaen-11-ol, aceto-cinnamone, cinnamyl-alcohol, and finally α-eudesmol and β-eudesmol. In the hills, instead, was found to be higher the hydrocarbons 2-butenal, 2-methyl- and 2-butenal, 3-methyl- as well as for two unknown sesquiterpenes (called sesquiterpene_3 and sesquiterpene_4). The PCA sub lining these differences is in Figure 2. 

Some of the compound mentioned above as characteristic of one or both the locations were also higher of 1% of total VOCs (Table 3). β-Linalool and cinnamyl alcohol were in fact present in a percentage higher than 1% only in plains samples. The most important volatiles with an amount that exceeded 1% of total VOCs for both Appennines and Plains were cinnamaldehyde, β-eudesmol and δ-cadinene. Aliphatic and aromatics alcohols, carbonyl compounds and aliphatic acids have been characterized among non-terpenes volatiles in a fraction of 280 µg/g for hills samples and 350 µg/g for the plains, while some compounds were not identified (about 70 µg/g for both hills and plains samples). A substantial amount of acids was found in the samples from both locations: Acetic acid, 2-methyl butanoic acid, 2-butenoic acid and 2-methyl propanoic acid and propanoic acid and α-methyl crotonic acid. The aromatic compounds such as benzaldehyde, benzyl acetate, benzyl alcohol and phenethyl alcohol constituted the significant amount of non-terpenoids VOCs fraction (Table 3). 

### 2.4. Nuclear Magnetic Resonance (NMR)

The NMR spectra of samples were recorded on a Bruker Avance spectrometer with proton operating frequency 600.13 MHz with a 5mm TBI probe. The spectra were performed at 300 K using 16 K of TD (time domain), acquisition time 1.27 min, delay time 1.0 s and 48 the number of scans. The spectral width was 12019 Hz. For ^1^H-NMR analysis 2–3 mg of crude extracts were dissolved in 0.6 mL of DMSO-d6, while for ^13^C-NMR spectra 10–20 mg for each sample were used.

**Figure 3:**
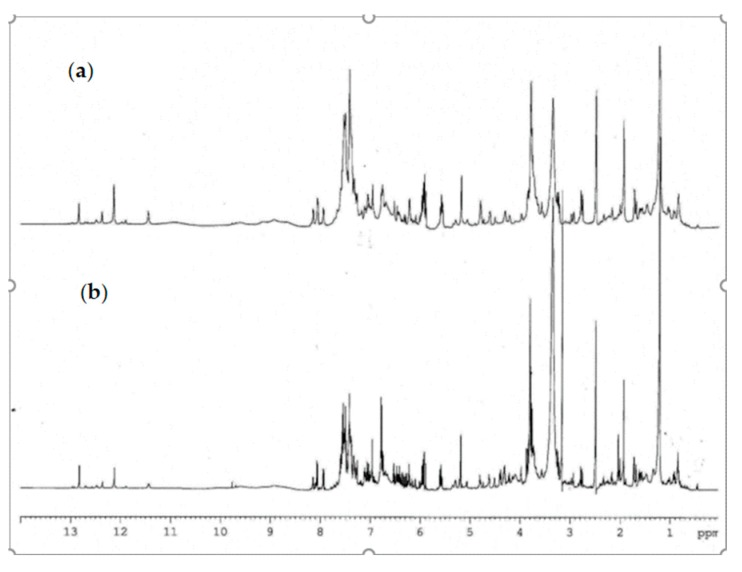
Comparison of ^1^H-NMR spectra of propolis extract (**a**) plain sample (**b**) hill sample.

The ^1^H-NMR analysis of complex matrix such as propolis extracts were complicated for the presence of a high number of similar compounds. A comparison of ^1^H-NMR spectra of propolis extracts is reported in Figure 3. The most interesting spectral region are between 3.50 and 8.25 ppm, which contains aliphatic and aromatic signals, and between 10.00 and 13.00 ppm which contains chelated phenolic groups and carboxylic proton signals. Figure 3 showed that the samples appear to be very similar to each other; the main signals associated with the secondary metabolites characterizing the extract appear to be present both in plain and hill samples, in some samples a slightly different quantitative ratio. Diagnostic signals related to chelated phenolic groups, typical of flavonoids, or hydroxyl groups of carboxylic functions can be detected at low fields between 12.0 and 13.3 ppm. The signals of the known compounds were determined by comparison of their physical and spectroscopic features with standard compounds and with those reported in the literature [34]. Variable amounts of flavonoid were identified: pinocembrin, chrysin, galangin, pinobanskin-3-*O*-acetate, and pinostrobin. Several phenolic acids, pinobanskin, kaempheride, apigenin, and other compounds were also present as minority components.

The phenyl ester of caffeic acid, known as CAPE, had the signals of the methylene protons resonating at 2.95 and 4.32 ppm; at the examined concentrations these signals were lacking and therefore CAPE was not detectable in our samples. Quercetin was not detectable in both extracts.

**Figure 4:**
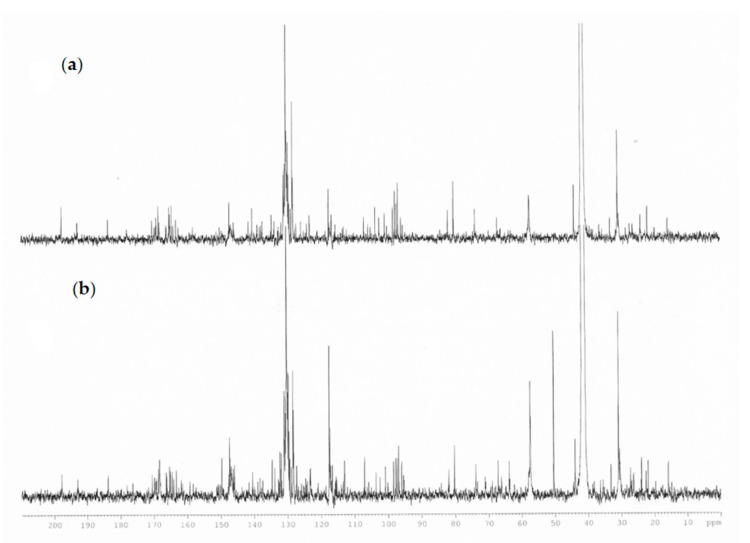
Comparison of ^13^C-NMR spectra of propolis extract (**a**) plains sample (**b**) hills sample.

Specific resonances attributable to glycerol esters (such as 1,3-di-*p*-coumaryl-2-acetyl-glycerol and 1,3-diferulyl-*p*-coumarate-glycerol) were given by the presence of signals in the zone 4.2–5.3 ppm (glycerol moiety), an area crowded with several overlapping signals, and a singlet resonance for methyl groups at 2.05 ppm. The presence of the acetyl groups and the ester groups were also confirmed by ^13^C-NMR spectrum due to the presence of signals in the area between 160 and 170 ppm, the portion of glycerol, instead, gives signals at 63–71 ppm, the methyl groups of acetyl at 19.7 and 21.9 ppm in agreement with the literature data (Figure 4) [25]. The definition of the type of glycerol ester was given with LC–MS orbitrap.

### 2.5. HPLC–Q-Exactive-Orbitrap®–MS Analysis

**Figure 5:**
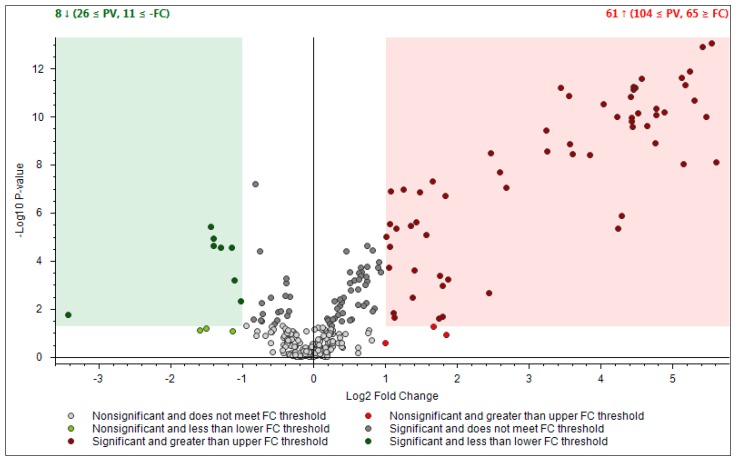
Differential analysis presented by Volcano Plot Model for the comparison between the relative intensity of chromatographic peak from two propolis groups. *P*-value (PV) was set on 0.05. Red region contains up-regulated signal where the quantities from Apennines was significantly higher than those found in Po Valley samples and was greater than the upper fold-change (FC) threshold. The green region comprises up-regulated peaks in Po Valley samples.

**Figure 6:**
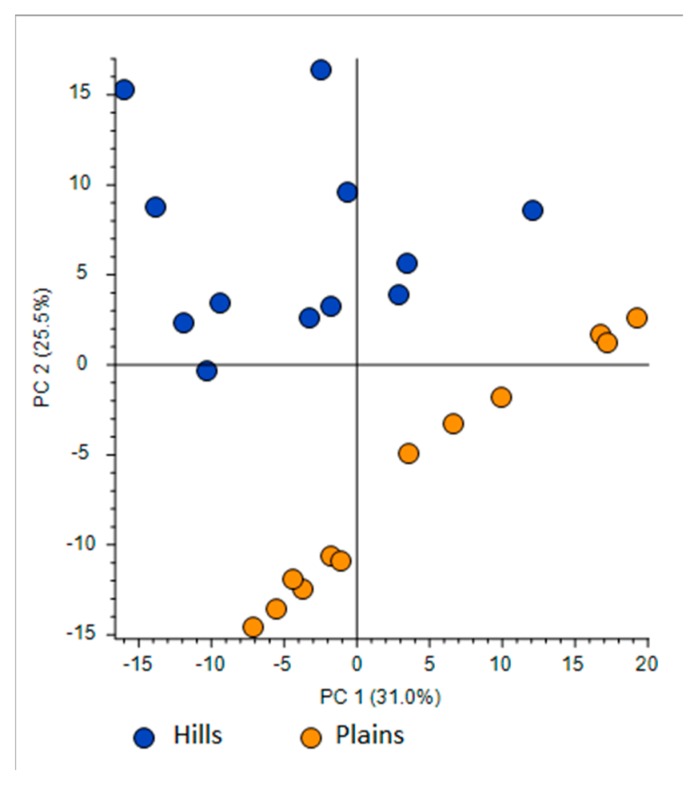
Principal Component Analysis (PCA) projection on the distribution of samples respect to PC-1 with PC-2 when high resolution mass spectrometry (HRMS) spectra were used.

Crude extracts that were used for HPLC–UV analysis were diluted (1:100) and were subsequently subjected to HPLC-Q-Exactive-Orbitrap*®*-MS analysis (activated in negative mode) in order to perform untargeted profiling of propolis collected from hills (Ligurian–Piedmont Apennines) and plain (Po Valley) with subsequent data processing performed by Compound Discoverer™ (CD) software. Two type of Q-Exactive-Orbitrap*^®^*-acquisition mode were executed:

- First one was full scan (FS) at maximum resolution of 140,000 that involved generation of the lists of compounds that are potentially present in the samples (307 candidates). Using Compound Discoverer platform compounds were identified applied workflow that includes RT alignment, blank subtraction, and molecular formula assignment. Also, in FS acquisition mode the additional detection settings were applied: (1) Selecting the unknown peaks with criteria such as mass tolerance (<2 ppm); (2) minimum peak intensity (100,000), 3) integrating isotope and adduct peaks of the same compound into one group to reduce the incidence of false positives. This phase involved also the differential analysis with Volcano Plot (VP) (Figure 5) and principal component analysis (PCA) (Figure 6). PCA clearly distinguished the hills and plains samples, where VP analysis gave more precise response which signals are the main contributors along with the statistical evaluation presented in Table 4.
- Second type of analysis regards FS-data dependent (FS-DDA) acquisition mode and was performed on the inclusion list of 307 signals extracted from the FS data collection. MS–MS fragmentation performed in FS–DDA modality enabled the putative identification beyond the available standards. This phase comprises molecular formula assignment according to the accurate mass, adduct state, isotopes and fragmentation patterns with selecting best-fit candidates for the non-target peaks after comparison and evaluation with the software-linked MS2 libraries (mzCloud, *m/z* Valut and ChemSpider). To make the results more reliable especially when the mzCloud did not give any well-defined response the matching results are further filtered and checked with other on-line databases (human Metaboloeme at the first place). In some cases, as we have not found any satisfactory confirmation from existing databases, the tentative deduction of the final structure was performed manually assigning the fragments structure in concordance with available literature [35,36].

Using the above described platform, it was possible to single out and speculate ninety compounds divided in the categories listed in the Table 4. Most phenols have been previously confirmed in the literature for “poplar type” propolis [35,36]. The method described allowed the hypothesis of the presence of some new compounds not previously found in propolis: 4-ethyl-7-hydroxy-3-(*p*-methoxyphenyl)-coumarin in plain samples and 4-hydroxy-4′-methoxychalcone in hills propolis. As showed in our study, the use of NMR analysis of complex matrix such as propolis extracts is complicated for the presence of a high number of similar compounds but further investigation with this and other analytical instruments should be effectuated to verify the presence of these new compounds. The most important results regard the strongly upregulated phenolic glycerides in hills samples. The main characteristic of hills samples was the unambiguous occurrence of different glycerol esters which HRMS signals were very poor in plains group. For the Po-Vally samples the two isomers of abscisic acid were dominant in samples from this area. Also, plains propolis revealed higher concentration of trans-cinnamic acid which is accompanied with its caffeic esters form. The Volcano Plot (Figure 5) segregated the selected metabolites as those responsible for grouping, while the PCA projection clearly demonstrated that same geographic regions are closely grouped, and the first two components describe 59% variability. 

## 3. Discussion

No significant differences were found between the two sampling sites for what concerns the balsam content. Also, no significative differences were found for total phenols, total flavones and flavonols and scavenging activity. This is probably due to the very low sensitivity of spectrophotometric evaluation, as the other analytical methods showed both quantitative and qualitative differences in the composition of the two propolis samples. As already reported in the literature [28], the difficulty in the measurement of antioxidant activity arises from the different standard reference compounds and there is a wide variety of methods to assess antioxidant capacity, each having advantages and disadvantages. For this aim, it would be important to find a standardized method to evaluate the antioxidant power of each kind of propolis, to valorize quality productions and avoid falsification. 

Moisture content was lower than 1% in all samples, as propolis is a material produced by bees to last long time in the beehive. 

VOCs determination can be considered an important aspect for propolis characterization [37]. The main influence on the aroma composition of propolis is formed by volatile compounds that may come from the material collected by bees and then it may largely depend on the plant of propolis origin. Other factors had been demonstrated, as the state of propolis maturity and the honeybees as well. Aldehydes and alcohols may also be a consequence of microbiological activity or heat exposure, while linear aldehydes are considered as characteristic compounds associated with certain herbal origin as terpenes and terpenoids. Acetic acid, hexanal, alpha pinene, camphene, benzaldehyde, octanal, nonanal, beta-ciclocitrale, cinnamaldeide, cinnamyl alcohol, alpha copaene, cinnamyc alcohol, acetocinnamone, cinnamic acid, gamma cadinene, guaiol, gamma and beta eudesmols, benzyl benzoate has been previously detected in propolis in [28] and the chemical composition of propolis volatile fraction determined in the present study by means of HS–SPME-GC–MS was found to be in agreement with previous reports [32,38].

Two of the compounds found significantly higher in the plains are α-eudesmol and β-eudesmol. The last is the most abundant compound in resins from leaf buds of black poplar (*Populus nigra* L.) [39], which represents one of the main botanical sources of propolis constituents in temperate regions [40]. Indeed, several volatile compounds identified in propolis volatile fraction have been previously detected from leaf buds of *Populus nigra*. As the *Populus nigra* is a plant that grows in perifluvial environments of the Po Valley [41], we can assume that the apiary in the plains found easier to collect resins from this plant. According to [32], propolis from temperate zones can be classified in two types, based on the presence of representative amounts of β-eudesmol (40%–60%) or benzyl benzoate (20%–40%) in the essential oil. In our research as well, the two considered type of propolis differs for eudesmol content. 

Likewise, flavonoid aglycones and esters of substituted cinnamic acids are the major constituents of propolis in the temperate zone where the basic plant source of bee glue are the bud exudates of trees of the genus *Populus*, mainly the black poplar *P. nigra*. [42]. In our study cinnamaldehyde, acetocinnamone, and cinnamyl alcohol were found significantly higher in the plain’s samples while the two unknown sesquiterpenes characterized the hills samples in absence of characteristic compounds of poplar exudates. This could be of interest because it has been shown that bees can find in their environment and use as propolis source the best agent to protect their hives against bacterial and fungal infections [43]. 

The acetic acid, a carboxylic acid, was found in high quantity in our propolis samples and significantly higher in the plains samples. Acetic acid was found in headspace volatiles (dynamic headspace sampling, DHS) of Chinese propolis from 23 regions of China, as one of the main aroma-active components [44]. SPME with GC–MS was used for analysis of volatiles of Chinese propolis from the Beijing and Hebei provinces and again acetic acid and phenethyl acetate were among the main volatile constituents, together with phenethyl alcohol [45]. Their composition was somewhat similar to the volatiles of gum from poplar growing in China [46]. So, also this compound confirms the poplar exudates as the main origin of propolis plant-based component. 

In our study we found low quantity of pinene (both in the plains and in the hills), that may be the consequence of collecting the resin of coniferous trees only when the other preferred sources are not sufficiently available [47]. This is probably due to the fact that conifers are scarce in the two sampling areas. 

Until now, the propolis from areas without black poplar has been very poorly investigated. Out of 114 propolis samples analyzed [24] only 17 originated from ‘‘northern and mountain groups’’. As it turned out, these samples contained considerably less (approximately 25%) biologically active polyphenols characteristic for ‘‘poplar type’’ propolis, ‘‘but did not have significantly lower antibacterial activity’’. If it can be assumed that in the composition of propolis not deriving mainly from poplar exudates, there must be some active substances of unknown origin and unidentified chemical structure [47].

With chromatographic condition applied herein it was possible to perform the unambiguous detection and subsequent identification of phenolic acids (caffeic, p-and m-coumaric, ferulic, *trans*-cinammic) as well as two flavonoids namely pinocembrin and chrysin. The other compounds that were afterward defined by HRMS and NMR analysis were not quantified due to matrix complexity. As our aim was to use a simple HPLC–UV run for the fast characterization of propolis, any modification of chromatographic/detector condition did not bring any improvement in separation of other propolis components. The HPLC–UV analysis confirmed the presence of a complex mixture of compounds; the chromatographic resolution was slightly improved to obtain the optimal conditions for HPLC–Q-Exactive-Orbitrap^®^–MS analysis. The HRMS analysis demonstrated the presence of phenolic acids, flavanones, flavones, chalcones and isoflavones in the composition of the plains and hills propolis. Plains propolis, as expected, reveled higher concentration of trans-cinnamic acids which is accompanied with its caffeic esters form, displaying the typical pattern of “poplar” propolis.

HPLC–Q-Exactive-Orbitrap*^®^*–MS and NMR showed to be complementary methods for propolis characterization as they confirmed the principal compounds. For for minor compounds, as CAPE, which was not detected by NMR, the technique of HPLC–Q-Exactive-Orbitrap*^®^*–MS was fundamental; on the other hand, NMR allowed the detection of of some very descriptive minor molecules as phenolic glycerides. In the hills’ samples, in fact, phenolic glycerides (dicoumaroyl acetyl glycerol, diferuloyl acetyl glycerol, feruloyl coumaroyl acetyl glycerol, caffeoyl coumaroyl acetyl glycerol) were upregulated. These compounds have been isolated [48] from North-Russian propolis and the exudate of *Populus tremula* L. (aspen) was found to be their plant source. Phenolic glycerides were previously detected in propolis samples from a mountain region at about 700 m a.s.l. in Switzerland where there are relatively high numbers of young *P. tremula* trees, and relatively few *P. nigra* [49].

*P. tremula* is present in the hills areas of the Ligurian–Piedmont Apennines. This species grows in abandoned fields in plant communities of *Sambuco-Salicion capreae* phytosociological alliance [50]. This vegetation is expanding both in the Apennines and in the Alps due to the drop out of agricultural practices [51]. Our study confirm that the determination of the “type” of propolis, according to its plant source, has to be the first step in quality control of bee glue and that bees have the ability to find in their environment and use as propolis source the best agent to protect their hives against bacterial and fungal infections [42]. 

It is also interesting the presence of abscisic acid in the Po Valley propolis samples. Abscisic acid is in fact a plant hormone with many functions, including seed and bud dormancy, the control of organ size and stomatal closure and it is sometimes involved in leaves abscission. The production of this and similar unusual compounds is a common ecological strategy in plants [52,53]. Therefore, the finding of high amounts of abscisic acid in the Po Valley samples is probably due to greater plant exposure to stress (dry, high temperature etc.) compared to those growing in the mountain areas.

So far, there is no evidence that individual propolis components are chemically modified by bee enzymes [54]. Our results support this finding as the agreement between the fingerprints of Apennines and Po-Vally propolis was surprisingly good. This points toward the conclusion that the difference revealed in relative amount of some components between two geographically different propolis sampling are due to botanical (vegetation) surrounding where bees collected the propolis.

The obtained results revealed importance of combined approach in analysis of complex biological matrices which composition can vary significantly depending on environmental conditions where is produced.

Propolis knowledge has registered an important evolution over time, due to exhaustive studies regarding its chemical composition and biological activities. In the 60’s, it was thought that, despite its complexity, propolis chemical composition was more or less constant [55]. Spectrophotometric analysis was thought to be enough descriptive of this matrix and also now spectrophotometric analysis of total phenols, flavones and flavonols and antioxidant activity are widely used. 

In the last decades analysis of a large number of samples from different geographic origins revealed that chemical composition of propolis is highly variable and also difficult to standardize because it depends on factors such as the vegetation, season, and environmental conditions of the site of collection [55]. Different resin types were proposed: poplar propolis, birch, green, red, “Pacific,” and “Canarian” and also classification by propolis color (green, red, brown etc.) [56,57]. We demonstrated anyway that propolis from the same geographic area (Northern Italy) and of the same color (brown) differs significantly for many bioactive compounds. In fact, HPLC–Q-Exactive-Orbitrap*^®^*–MS analysis has been crucial in the appropriate recognition of evaluate number of metabolites, but also NMR itself could give more detailed information especially when isomeric compounds should be identified. However, for full metabolomics profiling combining single instrumental technique is indispensable in propolis characterization as identified metabolites belong to different chemical groups, so different analytical techniques are required. 

## 4. Materials and Methods

### 4.1. Sample Collection and Study Area

The samples of propolis were collected from a professional beekeeper conducting 170 beehives for the harvests and around 100/200 breeding nucleuses for the company comeback and the sale. Propolis is harvested using both mesh and scraping. In our study we consider only propolis harvested by mesh. Samples were collected in a randomized selection of six beehives in the plains (Po Valley) and six beehives on the Ligurian–Piedmont Apennines. Considering that there are many factors which influence the phytochemical composition of propolis, the samples that were subjected to our evaluation originate from the same bees’ strain and were harvested by same method. The only variable was the two different apiary geographical locations.

**Figure 7:**
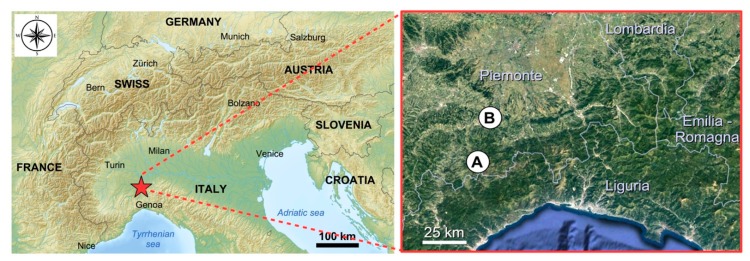
Localization of the two sampling areas: (**A**) hill (Ponzone); (**B**) plain (Visone).

The apiaries are sedentary and placed: (A) in the valley bottom, on the edge of the plain (municipality: Visone—AL; elevation: 100 m a.s.l.; Latitude: 44°35′21″ N; Longitude: 8°27′37″ E) and (B) on the hill (Municipality: Ponzone - AL; elevation: 550 m a.s.l.; Latitude: 44°39′46″N; Longitude: 8°30′06″E) and distant about 25 km (Figure 7). The two sampling areas belong to the “Alpi Marittime” Ecoregional Subsection of Italy (Western Alps Section, Alpine Province) [58] with “Temperate continental submediterranean” bioclimate [59]. The two sampling areas are different as regards their vegetation series (*sigmeta*). 

The sampling area A belong to *Physospermo cornubiensis-Querco petraeae sigmetum* where the mature stage of the vegetation series is the forest of *Quercus petraea* (dominant species) with other trees (*Castanea sativa*, *Sorbus aria*, *Fraxinus ornus*), shrubs (*Corylus avellana*, *Erica arborea*, *Frangula alnus*, *Juniperus communis*) and herbs (*Physospermum cornubiense*, *Pteridium aquilinum*, *Molinia arundinacea*, *Sesleria cylindrica*, *Carex montana*, *Euphorbia flavicoma*, *Brachypodium rupestre*) [59]. The main plants growing nearby the apiary and suited for honeybees’ visit are *Castanea sativa*, *Erica arborea*, *Calluna vulgaris*, *Genista pilosa*, *Populus tremula* and *Salix caprea.*

The sampling area B belong to the vegetation series of lower Po Valley where the mature stage is the forest of the *Carpinion betuli* phytosociological alliance with *Quercus robur*, *Carpinus betuli*, *Fraxinus excelsior*, *Tilia cordata* and *Robinia pseudoacacia*, this latter species is very common where anthropic disturbance is greater [60,61,62]. Moreover, the area B is close to the Bormida di Spigno river near which there is riparian vegetation with willows (*Salix alba*, *Salix eleagnos*, *Salix purpurea*) and poplars (*Populus nigra* and *Populus alba*) [63]. The main trees growing nearby the apiary and suited for honeybees’ visit are *Salix alba*, *Salix eleagnos*, *Salix purpurea*, *Populus nigra* and *Populus alba, Robinia pseudoacacia*, *Tilia cordata,* and *Ailanthus altissima.*

### 4.2. Extraction, Balsam and Moisture Content

Propolis was pulverized by freezing it at −80 °C for an hour and pounding it in a mortar. 

One gram of pulverized sample was weighed and dissolved in 30 mL of 70% ethanolic solution (70:30 ethanol:water) and stirred constantly for 2.5 h in a dark room at medium strength (200 rpm). The ethanol/water mixture (70/30) is the most commonly used extraction method for propolis as it is non-toxic and efficient in particular for polyphenols and flavonoids, responsible for the properties of the substance [24]. After that, ethanolic extract was separated by a 5 min centrifugation (5000 rpm at 5 °C) and the supernatant was separated from the residue by filtration (Whatman 3), as described in [21]. The supernatant was collected in a volumetric flask and topped up to 100 mL using the same 70% ethanol solvent. The final filtrates represent the balsam (tincture) of propolis and are referred to as PEE (propolis ethanolic extract). The yield was expressed as balsam content (soluble ethanolic fraction) and determined according to [24]. To this end, an aliquot (50.0 mL) of each ethanolic extract was evaporated to dryness on a rotary evaporator under reduced pressure at 40 °C.

The moisture content was determined as percentage weighting 1 g of propolis oven dried at 40 °C for 16 h.

### 4.3. Total Phenolic Content, Total Flavones and Flavonols, and Free Radical-Scavenging Activity

The ethanolic extract was diluted 1:10 to calculate the total phenolic content. The method used to determine the total phenolic content of the propolis extract was the one described in [21]. One hundred microliters of each extract of propolis plus 1900 μL distilled water were placed in a glass tube and then the solution was oxidized by adding 100 μL of FolinCiocalteau reagent. After exactly 2 min, 800 μL of 5% sodium carbonate (*w/v*) was added. This solution was maintained in a water bath at 40 °C for 20 min, and then the tube was rapidly cooled with crushed ice to stop the reaction. The generated blue color was measured using a spectrophotometer at 760 nm. In order to prepare the stock standard solutions, 25 mg of gallic acid or a were dissolved to a final volume of 25 mL methanol and stored at −20 °C. The calibration curve was carried out at the beginning of the working day and was prepared by appropriate dilution of each stock standard solution with 70% ethanol (y = 2.3454x + 0.0047; R^2^ = 0.9998). The ethanolic solution was used as a blank. 

The total flavones and flavonols (TFF) were estimated according to an aluminum chloride method following [63]. For the calibration curve, four standard solutions of quercetin in 80% ethanol (25, 50, 100, and 200 μg/mL) were prepared (y = 0.0099x – 0.055; R^2^ = 0.9999). A 0.5 mL portion of standard solutions was separately mixed with 1.5 mL of 95% ethanol, 0.1 mL of 10% AlCl_3_ in water (*w/v*), 0.1 mL of 1 M potassium-acetate, and 2.8 mL of 80% ethanol. After incubation at 20 °C for 30 min, the absorbance was measured at 425 nm. The 10% AlCl_3_ was substituted by the same quantity of distilled water in the blank sample. Similarly, 0.5 mL of each extract diluted to 1:50 (*v/v*) in 80% ethanol was analyzed as described above. The results are expressed as TFF% *w/w*.

DPPH radical scavenging activity measured with the method described in [64]. Fifty μL of various concentrations of propolis samples were added to 2 mL of 60 μM methanolic solution of 1,1-diphenyl-2-picrylhydrazyl (DPPH). Absorbance measurements were read at 517 nm, after 20 min of incubation time at room temperature (A1). Absorption of a blank sample containing the same amount of methanol and DPPH solution acted as the negative control (A0). The results are expressed as % inhibition of the free radical with DPPH, as described in [65].

### 4.4. HPLC Analysis 

The standards used: kaempferol, caffeic acid, p-coumaric acid, ferulic acid, *m*-coumaric acid, quercetin, trans-cinnamic acid, apigenin, genistein, chrysin, pinocembrin, formic acid, acetonitrile, and ethanol were purchased from Sigma-Aldrich (St. Louis, MO, USA). Gallic acid, Folin-Ciocalteau reagent and 1,1-diphenyl-2-picrylhydrazyl (DPPH) were also purchased from Sigma–Aldrich (St. Louis, MO, USA). All reagents and standards used were HPLC grade, and purified water from a Milli Q system was used throughout the experiments.

Individual stock solutions of each standard were prepared using ethanol for kaempeferol, pinocembrin, ferulic acid and m coumaric acid, DMSO and methanol (1:9) for quercetin, apigenin and chrysin, methanol for genistein, *p*-coumaric acid, caffeic acid and trans-cinnamic at 10 mg/mL, and stored at −20 °C. The working standard mixture solutions were made by diluting the appropriate amount of each stock standard solution to obtain 5 calibration levels (final concentrations of 31, 25, 62, 5, 125, 250, and 500 μg/mL).

The HPLC system used to determine the quantity of the most present phenols was a LC Agilent series 1200 (Waldbronn, Germany) consisting of a degasser, a quaternary gradient pump, an auto-sampler and a UV-Vis detector (Waldbronn, Germany). A Phenomenex Lichrospher C18, 4.6 × 250 mm, 5 µm column (Torrance, CA, USA) was used for this analysis with a column flow of 1 mL min^−1^. Sample injections were made at 10 µL for all samples and standards. The run time was 35 min, with 1 min post run time. Details about the method are as follows: column oven (20 °C); mobile phase A (0.05% formic acid); mobile phase B (acetonitrile); flowrate (1 mL/min); needle wash (100% acetonitrile); injection volume (10 μL); detection at 295 nm, 325 nm, and 375 nm. The gradient applied was: 0 min (15% B); 5 min (40% B); 25 min (50% B); 30 min (90% B); a low gradient between 40% and 50% B was used to separate the acid compounds. A blank injection was performed in all the trials to check chromatographic interference in the resolution. The retention times of all the standards were confirmed by individual standard injections. A fortification of random samples was used to check further the retention factors. A standard mixture to check the retention times was injected each working day. The samples were filtered through a 0.2 μm pore size membrane filter prior to chromatographic analysis. LOD (0.5 μg/mL) and LOQ (1 μg/mL) was calculated according S/N ratio 3 and 10, respectively. 

### 4.5. Solid Phase Microextraction (SPME) and Gas Chromatography Mass Spectrometry (GC–MS) Procedure

HS–SPME and GC–MS analysis were performed following the method in [28] opportunely modified. A 2 g amount of finely powdered raw propolis was weighed and put into 20 mL glass vials along with 100 μL of the IS (4-nonylphenol, 2000 μg/mL in 2-propanol). Each vial was fitted with a cap equipped with a silicon/PTFE septum (Supelco, Bellefonte, PA, USA). At the end of the sample equilibration time a conditioned SPME fiber was exposed to the headspace of the sample for 120 min using a CombiPAL system injector autosampler (CTC Analytics, Zwingen, Switzerland).

After sampling, the SPME fiber was immediately inserted into the GC injector and thermally desorbed. A desorption time of 1 min at 230 °C was used in the splitless mode. Before sampling, each fiber was reconditioned for 5 min in the GC injector port at 230 °C. 

Analyses were performed with a Trace GC Ultra coupled to a Trace DSQII quadrupole mass spectrometer (MS) (Thermo-Fisher Scientific, Waltham, MA, USA) equipped with an Rtx-Wax column (30 m × 0.25 mm i.d. × 0.25 µm film thickness) (Restek, Bellefonte, PA, USA). 

The identification was accomplished using computer searches on a NIST98 MS data library. In some cases, when identical spectra have not been found, only the structural type of the corresponding component was proposed on the basis of its mass-spectral fragmentation. If available, reference compounds were co-chromatographed to confirm GC retention times. The components of ethanol extracts of propolis were determined by considering their areas as percentage of the total ion current. Some components remained unidentified because of the lack of authentic samples and library spectra of the corresponding compounds.

### 4.6. NMR

The NMR spectra of samples were recorded on a Bruker Avance (Santa Barbara, CA, USA) spectrometer with proton operating frequency 600.13 MHz with a 5mm TBI probe. The spectra were performed at 300 K using 16K of TD (time domain), acquisition time 1.27 min, delay time 1.0 s and the number of scans 48. Was used a spectral width of 12019 Hz. For ^1^H-NMR analysis 2–3 mg of crude extracts were dissolved in 0.6 mL of DMSO-d6, while for ^13^C-NMR spectra 10–20 mg for each sample were used. 

### 4.7. HPLC–Q-Exactive-Orbitrap^®^–MS Analysis: Untargeted Metabolomics Approach

In order to perform HPLC–Q-Exactive-Orbitrap*^®^*-MS analysis, samples that were subjected to HPLC–UV analysis were diluted (1:100) in starting mobile phase. Chromatography was accomplished on an HPLC Surveyor MS quaternary pump, a Surveyor AS autosampler with a column oven and a Rheodyne valve with a 20 μL loop system (Thermo Fisher Scientific, San Jose, CA, USA). Analytical separation was carried out using a reverse-phase HPLC column 150 × 2 mm i.d., 4 μm, Synergi Hydro RP, with a 4 × 3 mm i.d. C18 guard column (Phenomenex, Torrance, CA, USA). The mobile phase was run as a gradient that consisted of water and methanol both acidified with 0.1% formic acid. The gradient (flow rate 0.3 mL/min) was initiated with 80% eluent 0.1% aqueous formic acid with a linear decrease up to 5% in 30 min. The mobile phase was returned to initial conditions at 36 min, followed by a 9-min re-equilibration period. The The column and sample temperatures were 30 °C and 5 °C, respectively. The mass spectrometer Thermo Q-Exactive Plus (Thermo Scientific, San Jose, CA, USA) was equipped with a heated electrospray ionisation (HESI) source. Capillary temperature and vaporizer temperature were set at 330 and 380 °C, respectively, while the electrospray voltage operating in positive was adjusted at 3.30 kV. Sheath and auxiliary gas were 35 and 15 arbitrary units, with S lens RF level of 60. The mass spectrometer was controlled by Xcalibur 3.0 software (Thermo Fisher Scientific, San Jose, CA, USA). The exact mass of the compounds was calculated using Qualbrowser in Xcalibur 3.0 software. The full scan (FS) with resolving power 140,000 in negative mode was used for the screening and statistical evaluation of obtained chromaptografic profiles. FS-dd-MS^2^ (full scan data-dependent acquisition) was used for confirmation. Resolving power of FS adjusted on 70,000 FWHM at *m/z* 200, with scan range of *m/z* 100–900. Automatic gain control (AGC) was set at 3e^6^, with an injection time of 200 ms. The AGC target was set to 2e^5^, with the maximum injection time of 100 ms. Fragmentation of precursors was optimised as three-stepped normalized collision energy (NCE) (20, 40, and 40 eV). Detection was based on retention time and on calculated exact mass of the protonated molecular ions, with at least one corresponding fragment of target compounds). Good peak shape of extracted ion chromatograms (EICs) for targeted compounds was ensured by manual inspection, as well.

Raw data from Xcalibur 3.0 software were processed with Compound Discoverer™ (Thermo Scientific, Waltham, MA, USA). In particular, this platform enables peak detection, retention time adjustment, profile assignment, and isotope annotation. A list of potential compounds was suggested for each chromatographic peak depending on the mass fragmentation of the parent pseudomolecular ion. Accurate mass determination generating elemental composition within a narrow mass tolerance window for identification based on accurate precursor mass. For some signals, the putative identification was confirmed by analysis performed on authentic standard. Compounds identification was based on accurate mass and mass fragmentation pattern spectra against MS–MS spectra of compounds available on mzCloud database (HighChem LLC, Bratislava, Slovakia). The ChemSpider Web services platform and Human metabolome [66] were used as additional confirmation tool. If mass fragmentation pattern did not correspond to any of databases annotated by Compound Discoverer™ software, manual confirmation using program ChemDrow of their fragments was performed. 

### 4.8. Statistical Analysis

The relative intensity of chromatographic peak from two propolis types were processed by Compound Discoverer platform that enabled differential analysis applying Volcano Plot Model and setting p-value (PV) on 0.05. In addition, the propolis samples were ordered by Principal Component Analysis (PCA) using HRMS spectra.

Data of moisture content, balsam content, total phenols, total flavones and flavonols, DPPH radical scavenging activity were analyzed using Student’s t- test at 95% confidence level in order to compare the two propolis types. The same statistical analysis was done for VOCs and single compounds quantified with HPLC. 

VOCs that resulted significant at the t-test were employed in the PCA to highlight the most important differences between the two batches of propolis type. T-test and PCA were performed using R 3.5.2 software [67].

## Figures and Tables

**Table 1:** Balsam and moisture content total antioxidant compounds and overall scavenging activity in raw propolis samples from hills (Ligurian–Piedmont Apennines) and plains (Po Valley).

Propolis Composition and Activity	Hills	Plains	Statistical Evaluation			
	Average ± SD	Average ± SD	*t*-Value	DF	*p*-Value	Sign.
Balsam content (% *w/w*)	75.92 ± 4.92	63.94 ± 12.86	2.131	6.433	0.074	ns
Moisture content (%)	0.74 ± 0.38	0.69 ± 0.52	0.195	9.233	0.850	ns
total Phenols (mg GAE/g)	242.42 ± 11.67	236.32 ± 40.92	0.351	5.808	0.738	ns
Total Flavones and Flavonols (mg QE/g)	32.14 ± 4.38	26.91 ± 4.31	2.082	9.998	0.064	ns
DPPH radical scavenging activity (%)	45.01 ± 1.39	46.44 ± 0.96	−2.082	8.855	0.068	ns

SD, standard deviation; ns, not significant; DS, degrees of freedom.

**Table 2:** The content of phenolic acids /flavonoids (mg/g) evaluated by HPLC-UV in alcoholic extract of propolis samples from hills (Ligurian–Piedmont Apennines) and plains (Po Valley).

Phenolic Acids/Flavonoids	Hills	Plains	Statistical Evaluation
	Average ± SD	Average ± SD	*p*-Value
caffeic acid	4.37 ± 0.53	4.21 ± 0.80	ns
*p*-coumaric acid	6.97 ± 2.12	1.40 ± 0.37	0.0001
ferulic acid	7.41 ± 2.22	1.64 ± 0.30	0.0013
*m*-coumaric acid	3.72 ± 0.27	2.87 ± 0.66	0.0150
*trans*-cinnamic acid	3.42 ± 0.21	4.48 ± 1.01	0.0428
pinocembrin	19.06 ± 6.27	17.90 ± 4.20	ns
chrysin	33.62 ± 3.49	35.64 ± 12.71	ns

SD, standard deviation; ns, not significant.

**Table 3:** Volatile compounds identified in raw propolis samples from hills (Ligurian–Piedmont Apennines) and plains (Po Valley).

RT ^a^	Compounds	Hills		Plains		*t*-Value	DF	*p*-Value	Signif. Code
		Mean^b^ ± SD ^c^	% ^d^	Mean ^b^ ± SD ^c^	% ^d^				
2.33	methyl-acetate	22.85 ± 5.11	5.52	34.23 ± 4.85	6.75	−3.9538	9.9717	0.002729	**
2.93	2,4−dimethyl-1-heptene	6.55 ± 0.83	1.58	10.41 ± 3.25	2.06	−2.8173	5.6514	0.03251	*
3.23	methyl-propanoate	0.69 ± 0.16	0.17	1.10 ± 0.28	0.22	−3.1264	8.1668	0.01373	*
6.00	α-pinene	0.55 ± 0.31	0.13	0.87 ± 0.46	0.17	−1.448	8.7216	0.1826	ns
7.23	3-buten-2-ol, 2-methyl-	1.33 ± 0.41	0.32	1.30 ± 0.36	0.26	0.14423	9.793	0.8882	ns
7.77	camphene	1.92 ± 0.55	0.46	3.53 ± 1.81	0.70	−2.0744	5.9194	0.08402	ns
8.98	esanal	2.96 ± 0.73	0.71	3.86 ± 1.59	0.76	−1.2607	7.039	0.2476	ns
9.24	2-butenal, 2-methyl-	1.15 ± 0.32	0.28	0.67 ± 0.13	0.13	3.4176	6.6211	0.01217	*
11.03	unknown_1	4.49 ± 0.52	1.08	4.40 ± 1.33	0.87	0.15257	6.4759	0.8834	ns
14.34	2-butenal, 3-methyl-	3.76 ± 0.85	0.91	2.68 ± 0.46	0.53	2.7426	7.6617	0.0264	*
14.76	unknown_2	7.04 ± 2.87	1.70	6.09 ± 1.75	1.20	0.69089	8.2804	0.5085	ns
16.70	unknown_3	19.67 ± 3.08	4.75	15.30 ± 6.80	3.02	1.4333	6.9764	0.195	ns
16.95	unknown_4	5.71 ± 2.21	1.38	5.48 ± 1.72	1.08	0.20213	9.4253	0.8433	ns
19.04	unknown_5	8.61 ± 1.21	2.08	12.20 ± 2.99	2.41	−2.7322	6.5943	0.03101	*
20.98	nonanal	1.92 ± 0.49	0.46	2.18 ± 0.35	0.43	−1.0457	9.071	0.3228	ns
21.32	benzene, 1-methoxy-2-methyl-	0.34 ± 0.12	0.08	0.93 ± 0.84	0.18	−1.7233	5.2184	0.143	ns
21.43	tetradecane	0.53 ± 0.13	0.13	0.65 ± 0.23	0.13	−1.1373	7.9121	0.2887	ns
21.74	2-octenal	0.50 ± 0.19	0.12	0.53 ± 0.20	0.10	−0.22144	9.9454	0.8292	ns
22.17	acetic acid	45.28 ± 6.83	10.93	57.80 ± 17.65	11.42	−1.6215	6.465	0.1525	ns
22.76	terpene_1	0.73 ± 0.26	0.18	2.46 ± 1.02	0.49	−4.0402	5.6519	0.007701	**
22.82	*trans*-linalool oxide	1.74 ± 0.50	0.42	1.61 ± 0.44	0.32	0.46595	9.8211	0.6514	ns
23.33	α-copaene	2.29 ± 0.94	0.55	1.98 ± 0.50	0.39	0.69802	7.6553	0.5058	ns
23.61	(+)-camphor	1.44 ± 0.30	0.35	2.89 ± 1.62	0.57	−2.1594	5.3419	0.07974	ns
23.84	benzaldehyde	11.11 ± 2.69	2.68	18.05 ± 4.59	3.57	−3.1941	8.0829	0.01255	*
24.23	propanoic acid	6.34 ± 1.30	1.53	9.04 ± 4.21	1.79	−1.5016	5.938	0.1844	ns
24.66	β-linalool	3.06 ± 1.34	0.74	6.70 ± 3.19	1.32	−2.5733	6.7075	0.03821	*
24.91	2-methyl-propanoic acid	19.88 ± 3.52	4.80	27.67 ± 11.59	5.47	−1.5762	5.9158	0.1668	ns
25.69	sesquiterpene_1	0.44 ± 0.08	0.11	0.42 ± 0.09	0.08	0.49589	9.7629	0.6309	ns
25.99	β-cyclocitral	3.80 ± 1.16	0.92	3.37 ± 1.41	0.67	0.57503	9.6507	0.5784	ns
26.17	unknown_6	16.14 ± 1.92	3.90	15.86 ± 4.33	3.13	0.14412	6.8858	0.8895	ns
27.03	2-methyl-butanoic acid	20.72 ± 3.90	5.00	28.79 ± 11.02	5.69	−1.6923	6.2338	0.1397	ns
27.35	2-butenoic acid	5.39 ± 1.52	1.30	10.50 ± 4.88	2.08	−2.4501	5.9572	0.05	ns
27.51	sesquiterpene_2	2.38 ± 0.82	0.57	1.72 ± 0.57	0.34	1.6229	8.9525	0.1392	ns
28.19	benzyl-acetate	8.76 ± 2.10	2.12	8.09 ± 2.47	1.60	0.506	9.7442	0.6241	ns
28.29	polycyclic aromatic compound	3.55 ± 0.54	0.86	2.69 ± 0.59	0.53	2.6531	9.9141	0.02435	*
28.84	δ-cadinene	13.63 ± 3.88	3.29	12.45 ± 3.63	2.46	0.54221	9.9557	0.5996	ns
28.99	unknown_7	3.25 ± 0.61	0.79	4.26 ± 1.89	0.84	−1.2451	6.0414	0.2592	ns
29.07	unknown_8	2.51 ± 1.40	0.61	3.46 ± 1.23	0.68	−1.2557	9.8345	0.2343	ns
29.25	unknown_9	1.50 ± 0.25	0.36	1.72 ± 0.59	0.34	−0.85269	6.6761	0.4234	ns
29.55	pentanoic acid, 4-methyl-	1.42 ± 0.56	0.34	1.27 ± 0.45	0.25	0.50394	9.5007	0.6258	ns
29.81	unknown_10	1.24 ± 0.51	0.30	1.57 ± 0.45	0.31	−1.1763	9.8542	0.2671	ns
30.18	calamenene	4.97 ± 1.45	1.20	4.01 ± 1.39	0.79	1.1844	9.9827	0.2637	ns
30.28	α-methyl crotonoic acid	43.69 ± 6.73	10.55	59.28 ± 16.62	11.72	−2.1297	6.5967	0.07309	ns
30.90	benzyl-alcohol	48.04 ± 10.13	11.60	38.98 ± 10.75	7.70	1.5022	9.9647	0.1641	ns
31.54	phenethyl-alcohol	24.41 ± 3.79	5.90	31.67 ± 7.52	6.26	−2.1124	7.3894	0.07045	ns
32.81	cinnamaldehyde	4.98 ± 0.89	1.20	9.57 ± 2.63	1.89	−4.0482	6.1243	0.006458	**
32.88	α-copaen-11-ol	0.74 ± 0.48	0.18	1.50 ± 0.48	0.30	−2.7538	9.9998	0.02034	*
33.04	octanoic acid	0.38 ± 0.11	0.09	0.54 ± 0.20	0.11	−1.7018	7.941	0.1275	ns
33.08	sesquiterpene_3	1.16 ± 0.43	0.28	0.22 ± 0.07	0.04	5.3763	5.2743	0.002547	**
33.15	sesquiterpene_4	1.69 ± 0.50	0.41	0.62 ± 0.22	0.12	4.8115	6.8895	0.002028	**
33.39	guaiol	0.39 ± 0.46	0.09	0.37 ± 0.12	0.07	0.12043	5.6469	0.9083	ns
33.56	acetocinnamone	1.14 ± 0.21	0.27	2.22 ± 0.74	0.44	−3.4706	5.8256	0.01394	*
34.11	unknown_11	0.78 ± 0.28	0.19	0.88 ± 0.23	0.17	−0.70911	9.6282	0.4951	ns
34.22	Sesquiterpene_5	3.04 ± 1.68	0.73	3.26 ± 0.55	0.64	−0.30368	6.0741	0.7715	ns
34.30	Sesquiterpene_6	1.19 ± 0.68	0.29	1.91 ± 0.58	0.38	−1.965	9.7885	0.07841	ns
34.40	Sesquiterpene_7	1.88 ± 0.99	0.45	1.97 ± 0.30	0.39	−0.21625	5.9323	0.836	ns
34.79	α-eudesmol	2.05 ± 0.93	0.49	4.49 ± 0.72	0.89	−5.105	9.399	0.00056	**
34.90	β-eudesmol	4.21 ± 0.68	1.02	8.42 ± 1.32	1.66	−6.9394	7.5091	0.000161	**
35.16	α-Copaen-11-ol	0.52 ± 0.47	0.13	0.79 ± 0.34	0.16	−1.1297	9.1588	0.2873	ns
35.60	cinnamyl alcohol	1.64 ± 0.35	0.40	5.39 ± 1.90	1.06	−4.7549	5.3445	0.004271	**

^a^ RT, retention time (min); ^b^ Mean, mean value (*n* = 6); data are expressed in µg/g; ^c^ SD, standard deviation (*n* = 6); ^d^ %—percentage of total VOCs; ^d^ Signif. Code, * *p* < 0.05; ** *p* < 0.01; ns, not significant.

**Table 4:** Tentative identification of compounds based on Compound Discoverer evaluation.

Compounds	Ret. Time	Formula	Exact Mass	Differential Analysis *
Phenolic acids and their derivatives				
*p*-hydroxybenzoic acid	5.48	C_7_H_8_O_4_	137.0244	ns
cinnamic acid isomer	5.87	C_9_H_8_O_2_	147.0452	up-regulated in hills
Salicylic acid	9.92	C_7_H_8_O_4_	137.0244	ns
Caffeic acid	10.52	C_9_H_8_O_4_	179.035	ns
*m*-Coumaric acid	11.03	C_9_H_8_O_3_	163.0401	up-regulated in hills
*p*-Coumaric acid	12.61	C_9_H_8_O_3_	163.0401	up-regulated in hills
Cinnamic acid	13.12	C_9_H_8_O_2_	147.0452	up-regulated in plains
Ferulic acid	13.43	C_10_H_10_O_4_	193.0506	up-regulated in hills
Cinnamic acid isomer	13.81	C_9_H_8_O_2_	147.0452	up-regulated in hills
Isoferulic acid	15.22	C_10_H_10_O_4_	193.0506	ns
Cinnamic acid isomer	16.15	C_9_H_8_O_2_	147.0452	up-regulated in hills
*p*-Coumaroylquinic acid	17.12	C_16_H_18_O_8_	337.0922	ns
Chlorogenic acid	21.05	C_16_H_18_O_9_	353.0876	ns
Benzyl-caffeate	21.17	C_16_H_13_O_4_	269.0819	ns
Prenyl-caffeate	21.7	C_14_H_16_O_4_	247.0976	up-regulated in hills
Caffeic acid phenethyl-ester (CAPE)	21.93	C_17_H_16_O_4_	283.0976	ns
*p*-Coumaric acid prenyl-ester	22.08	C_14_H_16_O_3_	231.1027	up-regulated in hills
Coniferyl-ferulate isomer	22.61	C_20_H_20_O_6_	355.1187	ns
Caffeic acid cinnamyl-ester	22.93	C_18_H_16_O_4_	295.0976	up-regulated in plains
Dupunin (Prenylated phenyl-propanoic acid)	23.01	C_14_H_16_O_3_	231.1027	ns
Coniferyl ferulate	25.51	C_20_H_20_O_6_	355.1187	ns
Capillartemisin A (Prenylated phenyl-propanoic acid)	25.74	C_18_H_24_O_4_	315.16	up-regulated in hills
Phenolic glycerides				
Caffeoyl-glycerol	10.01	C_12_H_14_O_6_	253.0713	highly up-regulated in hills
Dicaffeoyl-acetyl-glycerol	19.14	C_23_H_21_O_10_	457.1142	highly up-regulated in hills
Diferuloyl-glycerol	19.33	C_23_H_24_O_9_	443.1349	highly up-regulated in hills
acetyl-caffeoyl-feruloyl-glycerol	20.09	C_24_H_24_O_10_	471.1298	highly up-regulated in hills
Coumaroyl-caffeoyl-acetyl-glycerol	20.21	C_23_H_22_O_9_	441.1185	highly up-regulated in hills
Acetyl-coumaroyl-feruloyl-glycerol	21.17	C_24_H_24_O_9_	455.1347	highly up-regulated in hills
Di-*p*-coumaroyl-acetyl-glycerol	21.28	C_26_H_22_O_8_	425.1242	highly up-regulated in hills
Coumaroyl-acetyl-glycerol	24.22	C_18_H_16_O_3_	279.086	highly up-regulated in hills
Coumarins				
4-Ethyl-7-hydroxy-3-(*p*-methoxyphenyl)-coumarin	23.05	C_18_H_16_O_4_	295.0976	up-regulated in plains
Flavanones				
Pinostrobin	23.65	C_16_H_14_O_4_	269.0819	up-regulated in plains
Strobopinin	19.55	C_16_H_14_O_4_	269.0819	up-regulated in plains
Pinocembrin	21.88	C_15_H_12_O_4_	255.0663	ns
Sakuranetin	21.46	C_16_H_14_O_5_	285.0768	ns
3′,5,7-Trihydroxy-4’-methoxyflavanone	19.15	C_16_H_14_O_6_	301.0718	up-regulated in hills
Hesperetin	20.01	C_16_H_14_O_6_	301.0718	up-regulated in hills
Chalcones				
Pinostrobin-chalcone	19.62	C_16_H_14_O_4_	269.0819	up-regulated in hills
4-Hydroxy-4′-methoxychalcone	23.02	C_16_H_14_O_3_	253.087	highly up-regulated in hills
*Flavonols*				
Quercetin	18.2	C_5_H_10_O_7_	301.0354	ns
Quercetin-3-*O*-methyl-ether	19.01	C_16_H_12_O_7_	315.051	up-regulated in hills
Rhamnetin	20.16	C_16_H_12_O_7_	315.051	up-regulated in hills
Kaempferol	19.86	C_15_H_10_O_6_	285.0405	up-regulated in hills
Isorhamnetin	21.24	C_16_H_12_O_7_	315.051	up-regulated in hills
Kaempferide	22.93	C_16_H_12_O_6_	299.0563	up-regulated in hills
Bis-methylated quercetin	21.79	C_17_H_14_O_7_	329.0667	ns
Galangin	23.87	C_15_H_10_O_5_	269.0455	ns
Rhamnocitrin	23.18	C_16_H_12_O_6_	299.0563	up-regulated in hills
Flavanonols				
Pinobanksin	18.05	C_15_H_12_O_5_	271.0613	ns
Pinobanksin-5-methyl-ether	17.59	C_16_H_14_O_5_	285.0768	ns
Pinobanksin-5-methylether-3-*O*-acetate	20.2	C_18_H_16_O_6_	327.0869	up-regulated in hills
Pinobanksin-3-*O*-propionate	22.78	C_18_H_16_O_6_	327.0869	up-regulated in hills
Pinobanksin-3-*O*-butyrate	24.02	C_19_H_18_O_6_	341.103	ns
Pinobanksin-3-*O*-acetate	21.43	C_17_H_14_O_6_	313.0712	ns
Aromadendrin	14.85	C_15_H_12_O_6_	287.0651	ns
Isoflavones				
Genistein	19.08	C_15_H_10_O_5_	269.0542	ns
Formononetin glucoside	18.28	C_22_H_22_O_9_	429.1191	highly up-regulated in hills
Formononetin (biochanin B)	20.8	C_16_H_12_O_4_	267.0663	ns
Hispiludin	20.52	C_16_H_12_O_6_	299.0563	up-regulated in hills
Flavones				
Apigetrin (Apigenin-7-*O*-glucoside)	18.01	C_21_H_20_O_10_	431.0983	highly up-regulated in hills
Apigenin	20.44	C_15_H_10_O_5_	269.0542	up-regulated in hills
Dihydroxyflavone	21.43	C_15_H_10_O_4_	253.0506	ns
Chrysin	22.04	C_15_H_10_O_4_	253.0506	ns
Methoxy-chrysin	23.01	C_16_H_12_O_5_	283.0612	ns
Tricin	20.59	C_17_H_14_O_7_	329.0667	ns
Chrysoeriol	20.52	C_16_H_12_O_6_	299.0563	up-regulated in hills
Terpenoids				
Ursolic acid	29.65	C_30_H_48_O_3_	455.3531	highly up-regulated in hills
*trans*,*trans*-Abscisic acid	16.58	C_15_H_20_O_4_	263.1289	up-regulated in plains
*cis*,*trans*-Abscisic acid	23.66	C_15_H_20_O_4_	263.1289	up-regulated in plains
Unknowns				
Unknown 1 (phenylacetaldehide or isomer)	12.51	C_8_H_8_O	119.0502	highly up-regulated in hills
Unknown 2 (*p*-coumaric derivate)	14.97	C_19_H_20_O_6_	359.11	highly up-regulated in hills
Unknown 3 (ferulic acid derivative)	15.01	C_20_H_22_O_8_	389.124	highly up-regulated in hills
Unknown 4	18.28	C_27_H_42_O_4_	429.1191	highly up-regulated in hills
Unknown 5	19.05	C_17_H_16_O_6_	315.0851	highly up-regulated in hills
Unknown 6	19.11	/	597.1007	highly up-regulated in hills
Unknown 8 (*p*-coumaric acid derivative)	20.05	C_28_H_27_O_7_	475.1762	ns
Unknown 9 (Chrysin derivate)	21.09	/	639.1112	highly up-regulated in hills
Unknown 10 (*p*-Hydroxybenzoic acid derivative)	21.15	C_28_H_27_O_7_	475.1762	ns
Unknown 11	21.19	/	461.1007	highly up-regulated in hills
Unknown 12	22.29	C_20_H_37_O_9_	421.2441	highly up-regulated in hills
Unknown 13 (flavone ester of caffeic acid)	22.78	/	565.1509	up-regulated in plains
Unknown 14 (Chrysin derivate)	23.44	C_25_H_22_O_6_	417.135	highly up-regulated in hills
Unknown 15 (Chrysin derivate)	24	C_25_H_22_O_6_	417.135	highly up-regulated in hills
Unknown 16	25.17	/	413.1963	highly up-regulated in hills
Unknown 17	26.5	C_18_H_30_O_3_	293.212	highly up-regulated in hills

* Differential analysis performed applying Volcano Plot Model (Figure 5); ns, not significant.
